# Rutgers Medical College; an Introductory Lecture Delivered at the College of Physicians in the City of New-York

**Published:** 1830

**Authors:** John B. Beck

**Affiliations:** Professor of Materia Medica and Medical Jurisprudence in the University of the State of New-York


*Rutgers Medical College. An introductory lecture, delivered at the College of Physicians and Surgeons of the City of New-York, November, 6, 1829.* By JOHN B. BECK, M. D., *Professor of Materia Medica and Medical Jurisprudence in the University of the State of New-York.*

It was the wish of the individual whose name is appended to these remarks to have published something of the same character more than a year since, but the Editor at that time declined to admit them, from an unwillingness to involve himself in the controversies of distant and rival institutions. But in discussing the propriety and expediency of legislative interference to keep down rivalry among literary institutions, we are contending for an important principle in which every state of the Union has a common interest, and consequently, cannot subject ourselves to the imputation of taking part in controversies which do not belong to us.

Most of our readers, we presume, are aware of the circumstances under which Rutgers Medical College was established. A few years since, the professors in the Medical Department of the University of New-York, in consequence of a difficulty with the Regents, resigned their seats, and established a rival institution under the auspices of Rutgers College in New-Jersey. Laws founded upon the circumstance of the degrees being conferred by a Board resident in another state, having been passed by the Legislature with the design of preventing the Graduates of the New School from practising Medicine in the State of New-York, the institution was placed under the auspices of Geneva College. In the spring of 1829, the Professors applied to the State for a charter, and a bill for that purpose passed the Senate by a majority of four-fifths of that body, but not receiving the requisite majority of two thirds in the lower House, it failed to become a law. Since that time, we learn from the Newspapers, that the Supreme Court of that State, on a writ of *quo warranto*, have decided that the Trustees of Geneva College have no right to confer degrees upon the students of Rutgers Med. College.

Our attention has been again called to the subject by meeting with the Introductory Lecture of Dr. Beck—a production so far as style is concerned, every way worthy of the high reputation of the author. But as our opinions differ very widely from his, and as our prejudices in favor of R. College are very strong, we regret that our limits permit us to extract only the conclusion of the address in justification of the strictures we feel ourselves called upon to make.

But not merely in this respect does this college challenge an advantage. By the constituted authorities of our state, she has always been viewed with special regard, and the honours conferred upon her graduates are of a peculiar value. Her diplomas emanating from the regents of the university, are strictly university honours, and not mere college ones. And in receiving them, the name of every candidate is made known to a body of illustrious men, identified with the literary and political history of our country.

All this, however, would be of little avail, were not the honours and privileges emanating from her, of an enduring character. History, not very remote, furnishes us with the melancholy instances of colleges, struggling through a feverish existence of a few years, which, either from some defect in their corporate powers, or some other equally fatal cause, have been blotted from existence, leaving not a single memorial behind them. To the reflections of its alumni, such a fate cannot be otherwise than distressing. Every man of honourable feelings is deeply attached to the alma mater where he received his education. There he first became conscious of the extent and power of his capacities. There his mind was formed to habits of thought and study. There he received the first impulses to high and noble achievement. There he formed associations which have swayed the whole destiny of his after life. Loaded with honours, he leaves her with regret. But he can never forget her. Amid the busy scenes of life, his purest thoughts are directed towards her ; and in the wane of manhood, as years steal over him, he enjoys a melancholy pleasure in gathering up the early recollections of his collegiate career. How embittered must all these recollections be, should some un-toward fortune have prostrated in the dust, the institution with which they are associated. When the Romans denounced their heaviest imprecation upon the person who should destroy the monument of his ancestors, they merely wished that he might outlive all his relatives and friends, supposing this to be the the greatest curse that could befal him. Quisquis hoc sustulerit aut jusserit, *ultimus moriatur*. Something like this must be the situation of one who finds himself outliving the institution from which he received all his literary or professional honours. Such a fate can never befall the graduate of this college. She has already stood the test of time. Safe in her rights, every year but adds to her security and multiplies her triumphs. And in the successive classes which are annually resorting hither, distinguished for their talents, industry, and enthusiasm, I witness the evidence and the pledge of her present safety and her future prosperity.

It is always painful to see great and ingenuous minds, attacking a rival—a rival too that is struggling with adverse circumstances, by arguments addressed to sentiments and prejudices that can have no place in a well regulated mind. A diploma from the university derives its whole value from the names of the professors attached to it; any consequence it may receive from the university is altogether factitious, arising from the proneness of mankind to attach more importance to the name than the substance of things. Can the mere idea of a mechanical unintellectual corporation, chartered for conferring degrees, compare for one moment with the character and influence of the distinguished individuals at the head of the new Medical School. If this species of sentimentalism is entitled to any weight the advantages are entirely on the other side. There is an infinite pleasure in having a name attached to your diploma, which you are confident must be an object of admiration and envy so long as Surgery shall continue to be cultivated as a science, and long after the university and its rival School shall be forgotten. There is a pleasure also of a high order connected with the consciousness of having exerted an influence in favor of talents and merit, struggling against the influence of unjust and oppressive legislation, that is at least equal to the gratification of receiving literary honors from so distinguished an Alma Mater as the University of New-York.

It has fallen to our lot to have a full opportunity of observing the management and utility of collegiate instruction in the United States; and we have no hesitation in saying that its advantages are very generally misapprehended, and its importance entirely overrated. There are no schools which have been estimated so extravagently, and which do so little to meet the public expectations, as those of Medicine. Before the invention of the art of printing, when learning was confined to the halls of universities, the treasures of science could be obtained only through the medium of public lectures; but at the present time when the student, at an expense scarcely superior to that of a course of lectures, can collect in his study the united experience and observation of the profession, and can study, compare, and arrange at his leisure the opinions of the most distinguished men, a course of lectures can be useful only as subsidiary, and strictly subordinate to a well regulated plan of reading, and a judicious use of the other advantages which ought to be afforded by institutions of this kind. Lectures may be useful by establishing an elevated standard of professional excellence, by inspiring a love of fame and a professional feeling, by directing the labors of the student, and animating his progress; but the variety and extent of the sciences subsidiary to medicine are too great to admit of any plan of oral instruction supplying the place of patient investigation in the study, and unremitted labor in the field of observation.

It requires a well regulated mind, with a very considerable degree of information, to hear lectures with much advantage under any circumstances. But in the medical schools of this country, these things are managed in the worst possible manner. The great desideratum appears to be, to deliver the greatest possible number of lectures in the shortest time ; and consequently, the whole circle of medical literature is to be hurried through in the space of less than four months. That a person may form some idea of the imposition thus practised upon the world, let him imagine a young man, little acquainted with the principles of the profession, and still less with the habit of arranging his ideas with accuracy and order, to have arrived at the head quarters of Medical literature. Scarcely has he time to look around him before his arduous and unceasing labors commence. At 9 in the morning perhaps he hears an hours dissertation on the Materia Medica, in which pharmacy, chemistry, botany, and mineralogy are blended with Medical Jurisprudence and abstruse speculations on the *modus operandi* of medicines. The Institutes and Practice of Medicine follow, in which the nature, symptoms and treatment "of all the numerous ills that flesh is heir too" are detailed with scrupulous precision, and all the ridiculous speculations that have disgraced the profession for the last 2000 years, are called "up from the tomb of all the capulets" to undergo a serious refutation. Anatomy perhaps succeeds, and it is a subject of infinite humour, to see the professor surrounded by from 100 to 500 students, himself standing in a dark hole in the centre, demonstrating the minute anatomy of the eye or ear, or perhaps displaying some complicated structure, which the student must dissect repeatedly with great care before he can even name the parts when exposed fairly to his view ; and lecturing in the mean time, with the most inconceivable volubility, and with a multitude of the most uncouth and barbarous terms which the tongue was ever called upon to frame. The professor of Surgery follows; with equally favorable opportunities for the student to witness him cut among parts, where the deviation of the eighth of an inch in the point of the knife, would perhaps destroy life. In the afternoon come on chemistry and obstetricks, with perhaps a duplicate lecture or two on some one of the above subjects.

Exhausted and distracted by the laborious and varied mental exercise of the day, the student has the evening to recruit his spirits, to arrange and digest the information he has received during the day, and, if he has the pecuniary means, by his own labor to procure that anatomical knowledge, which ought to be his principal object in attending a course of Medical Lectures. Attendance on two courses of this routine of farce and humbug is called finishing an education, and next in order comes the diploma. And what does the diploma amount to ? An evidence that the gentleman thus dignified is qualified to practice medicine ? Nothing of the kind. Wealth may purchase the honor, the influence of friends may secure it, or dogged resolution in attending three or four courses of lectures will at length weary out the patience of the professors, and enable the veriest dunce in the Universe to carry off the prize. It amounts simply to shew that the persons who wear this distinguished honor have been able to raise the means to attend two courses of lectures.

This is a fair representation of that system of instruction that is pursued in every Medical College in the United States, a system that is supported at an expense of half a million of dollars annually—a system that is absurdly expected by a large portion of the community, to supply every defect of talents and industry, and transform men destitute of every qualification into accomplished physicians. We appeal to the public to say if it is not one of the greatest impositions ever palmed upon an enlightened age ; if it is not perfectly inadequate to the objects in view, and at least five centuries behind the present condition of literary improvement. Yet the gentlemen at the head of these institutions who have in consequence an opportunity of lecturing themselves into notice, and thereby of rising above professional competition, with all the gravity of a Roman Augur, talk sentimentally about the attachment of young men to their Alma Mater ; and from a generous attachment to the interests of literature, very modestly pray that they may be protected against competition.

A radical reform in this system is necessary, and as gentlemen who have enjoyed the advantages of these institutions, and derive much of their consequence from them will be backward in taking the necessary measures, we cannot conceive of any means so likely to be successful as the competition of rival schools, established in our large cities, whose professors may be urged by the pressure of *immediate and personal rivalry*, to place such advantages in the way of students, as will put down the petty establishments starting up in every corner of the country, where anatomy is taught without subjects, and the practice of medicine without patients.

To this we can see but two possible objections.

1st. That the standard of medical education may be lowered by the competition of numerous schools, striving to gain an ephemeral popularity by the facility with which they confer degrees. 2. That the competition will reduce the compensation of professors so low, that men of character and suitable qualifications will be unwilling to act in that capacity. These objections are not confirmed by experience.

1. The competition under a judicious system of instruction will probably be very little greater than it now is. Large cities alone afford the necessary opportunities for the advantageous study of medicine, and one of the most obvious results of rival schools in the cities, is to break up the country establishments. The advantages of state patronage are such that none but men of tried character can establish a rival college, and consequently the great danger is of too little, rather than too much competition.

2. High salaries eventually prove disadvantageous to literary institutions. When they become greater than a fair compensation for professional labor, the office becomes an object of intrigue, and other qualifications than talents, industry and ambition open the door to preferment. "When our school was poor" said a Leyden professor to an Edinburgh student, "scholars flocked to it from every part of Europe, but since it has been richly endowed, they have gone to seek teachers whom great wealth has not disqualified for the task of instruction."

3. It is a great disadvantage attending all literary institutions, that professors when once elected are allowed, by a kind of prescriptive right, to retain their seats whether they satisfy public expectation, or not. Let these institutions be subjected to a powerful competition, and some gentlemen who have been for years filling their pockets by lecturing to empty seats, or by lecturing the seats empty, will be sent out to make their living, in a more honest and honorable manner.

4. Lastly, one most important advantage attending a collegiate course of instruction is that young men are brought in close contact with great minds, whose intercourse inspires a kindred feeling of emulation and generous ambition. The monopoly of a state institution inevitably creates an aristocracy, that keeps the common herd of students at a distance ; but under a different state of things every student becomes a man of consequence, and every advantage is afforded to facilitate his labors, and animate him in his progress. F.

